# Systemic *Rasamsonia argillacea* infection in a German Shepherd dog

**DOI:** 10.1016/j.mmcr.2026.100779

**Published:** 2026-03-07

**Authors:** Louisa Schmidt, Sebastian Reusch, Ilka McCormick-Smith, Jasmin Gerkrath, Martin Peters, Volker Rickerts

**Affiliations:** a Fachtierarzt Hamm Dr. Lars Nethe, Marker Dorfstraße 132, 59071, Hamm, Germany; b Unit 16, Mycotic and Parasitic Agents and Mycobacteria, Robert Koch-Institute, Berlin, Germany; c Chemical and Veterinary Investigation Office Westphalia, Zur Taubeneiche 10-12, Arnsberg, 59821, Germany

**Keywords:** *Rasamsonia argillacea*, *Geosmithia argillacea*, Systemic mycosis, German shepherd

## Abstract

This case report describes a disseminated *Rasamsonia (R.) argillacea* infection in a German shepherd dog involving internal and external lymph nodes, abdominal organs and one eye. *R. argillacea* is a member of the *R. argillacea* species complex, which is an emerging pathogen notable for azole resistance in the last decades. The dog was euthanised following 18 days of itraconazole therapy due to clinical deterioration.

## 1 Introduction

*R. argillacea* is a fungal species complex with an increasing number of case reports in both human and veterinary medicine over the last 15 years. Overall, it is a rare fungal disease. It is considered an emerging pathogen, although the impact of increasing molecular diagnostics for species identification is unclear [[1], [2], [3], [4]]. A total of 24 cases of invasive fungal infection have been documented in human medicine between 2010 and 2023 [1,5] and hitherto 15 case reports have been published in the veterinary literature [[6], [7], [8], [9], [10], [11], [12], [13]]. In addition, respiratory colonization has been described in human medicine in patients with cystic fibrosis [3,4,14]. The main risk factors for invasive infection in humans are immunosuppressive conditions, such as chronic granulomatous disease (CGD) or immunosuppressive treatment [1]. 10 of the 15 veterinary cases were reported in German Shepherd dogs or German Shepherd crosses [[6], [7], [8], [9], [10], [11], [12], [13]]. This breed is thought to be immunocompromised, but the cause has not yet been identified [15]. In all cases infection was disseminated and often associated with discospondylitis. The overall prognosis is poor [[6], [7], [8], [9], [10], [11], [12], [13]].

*R. argillacea* is thermotolerant to thermophil. Microscopically, conidiophores have rough-walled stipes, conidia are olive-brown and ascomata, if present, have a scanty covering [16]. Because of phentotypical similarity to *Paecilomyces* spp. and *Penicillium* spp. this fungus may be misidentified [1,2,16,17]. The genus was reclassified in 2011 based on phenotypic, physiological and molecular characteristics [16]. Previous names were *Penicillium argillaceum* and *Geosmithia argillacea* (sexual state: *Talaromyces eburneus*) [2]. In addition to clinical samples, *R. argillacea* species have been identified in a range of other environmental samples, including mine tips, indoor air, soil, wood chips, plant seeds and as a contaminant of juice [2,18].

## 2 Case presentation

A 4-year-old female intact German shepherd dog was presented in a veterinary practice in January 2023 (day 0) due to vomiting, decreased appetite, polyuria and polydipsia. The dog received symptomatic, antiemetic therapy. On day +3, the dog came back due to insufficient improvement. On clinical examination the lymphonodi poplitei were slightly enlarged and a hyperthermia of 39.8 °C could be diagnosed. The abdominal sonographic examination showed highly enlarged lymph nodes with inhomogeneous parenchyma. A fine needle aspiration (FNA) sample was taken from one of the abdominal lymph nodes and submitted for cytological examination as well as a blood sample for haematological and blood chemistry analysis. Antibiotic and anti-inflammatory therapy with amoxicillin/clavulanic acid (16mg/kg q12h p.o.) and firocoxib (6.5mg/kg q24h p.o.) was started due to a possible bacterial infection causing the fever of unknown origin.

Blood results showed mild leukocytosis with absolute monocytosis, hyperphosphatemia, hypalbuminemia and hyperglobulinemia. Canine C-reactive protein (cCRP) was significantly elevated. Histopathological results of the FNA revealed pyogranulomatous inflammation with fungal hyphae (day +9). Systemic mycosis was suspected and a mycological culture was recommended.

On day +11 new enlarged lymph nodes (inguinal, axillary, superficial cervical) were found. The abdominal sonographic examination still revealed severely enlarged abdominal lymph nodes. The spleen showed multiple hypoechogenic, mostly small nodules with poor demarcation. The corticomedullary junction of both kidneys was less defined than normal and a small amount of ascites was present. Urinalysis showed haematuria and mild leucocyturia. The chest radiographs showed a low to moderate amount of pleural effusion. Enlarged mediastinal and sternal lymph nodes were suspected. Systemic fungal infections are generally rare in small animal medicine. *Aspergillus* spp. are the most frequently reported causative pathogens, so they were also suspected in this case. Systemic antimycotic treatment with itraconazole (6mg/kg q24h p.o.) was started and antibiotic treatment was stopped. A lymph node and a urine sample were submitted for mycological culture, but both showed no growth after 7 days of incubation.

The dog was presented again on day +24 with haemorrhagic diarrhoea. On clinical examination most of the external lymph nodes were still enlarged. Uveitis was suspected in one eye due to vitreous opacity. Abdominal ultrasound showed that the abdominal lymph nodes were still highly enlarged. The splenic parenchyma was less inhomogeneous than on day +11. Significant thickening of the gallbladder wall indicated cholecystitis. The owner declined further treatment. Despite additional therapy for haemorrhagic diarrhoea, combined with antimycotic therapy, the dog was euthanised on day +29 due to lack of improvement.

**Fig. 1:**
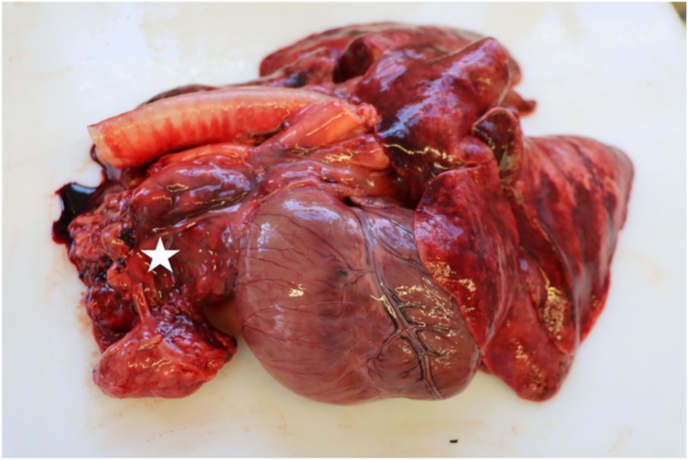
Severely enlarged cranial sternal lymph nodes (star) and mediastinal lymph nodes.

**Fig. 2:**
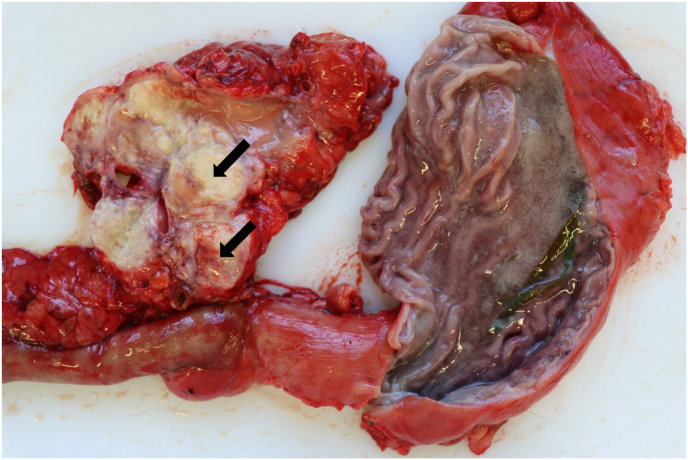
Granulomatous lymphadenitis of pancreatic lymph node (arrows).

**Fig. 3:**
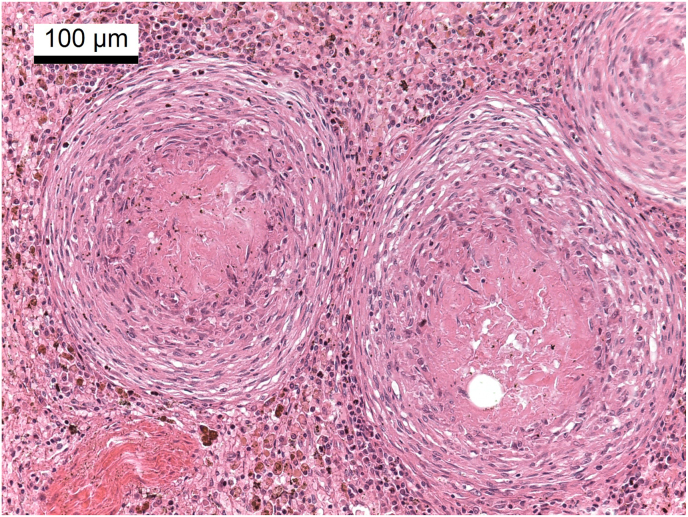
Spleen, histological section (HE stain): well demarcated granulomas with central necrosis surrounded by a narrow band of lymphohistiocytic cells.

**Fig. 4:**
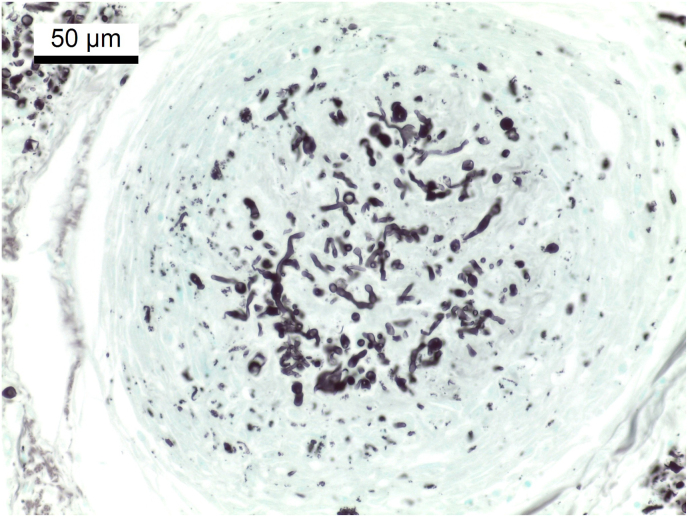
Spleen, histological section (Grocott's methenamine silver stain) showing pleomorphic fungal elements in the center of a granuloma.

Post mortem examination revealed multifocal pyogranulomatous lymphadenitis with enlargement of most external and internal lymph nodes. The sternal lymph nodes, bifurcation lymph nodes and pancreatic lymph nodes were particularly affected (Fig. 1). On cut surface, there were yellow nodules of up to 3 cm with soft centers (Fig. 2). Disseminated granulomas of the described morphology, but with fibroblastic encapsulation were present in liver, spleen, kidney and pancreas. Histologically, these granulomas showed central necrosis surrounded by a narrow band of lymphohistiocytic cells with few multinuclear giant cells (Fig. 3). Using Grocott's methenamine silver stain infiltrating septated fungal hyphae with some bulbous dilations were demonstrated (Fig. 4) The left eye revealed a purulent endophthalmitis and retinitis. Infiltrating hyphae could be demonstrated within the retina and vitreous body. The lungs and sinuses were free of signs of fungal infection.

Genus level identification was done by extracting DNA from the formalin fixed, paraffin-embedded (FFPE) lung, lymph node and spleen biopsies and fungal DNA was amplified by a broadrange PCR assay targeting the internal transcribed spacer (ITS) 2 region of the fungal ribosomal RNA genes. Sanger sequencing and a pairwise alignment search (https://its.mycologylab.org/page/Alignment) revealed *R*. *aegroticola* (100% pairwise identity), *R*. *argillacea* (100% pairwise identity) and *R. piperina* (95.88% pairwise identity) to be the closest matches for the ITS2 amplicons. To further identify the fungus on the species level, a second PCR assay targeting the fungal β-tubulin gene was performed from a fungal culture that was established post mortem from a lymph node biopsy. The amplicon was subjected to BLAST (https://blast.ncbi.nlm.nih.gov/Blast.cgi) and the search revealed a similarity to *R. argillacea* (98.99%), *R. piperina* (98.49%), *R. eburnea* (98.49%) and *R. aegroticola* (97.99%). Subsequently, a maximum likelihood phylogeny analysis with the ITS2 and β-tubulin amplicons was performed using the CLC genomics workbench (Qiagen). Phylogenetic analysis suggested that the dog was infected with *R*. *argillacea*. Antifungal susceptibility testing was performed using the broth microdilution reference method for filamentous fungi of the Clinical and Laboratory Standards Institute (CLSI). Testing revealed *in vitro* activity of Amphotericin B and Anidulafungin and reduced activity of tested azoles (see Table 1).

**Table 1:** Antifungal susceptibility test results of the *Rasamsonia spp*. isolate cultured from lymph node of the dog. AMB: Amphotericin B, ITZ: Itraconazole, PCZ: Posaconazole, VCZ: Voriconazole, AND: Anidulafungin, MIC: Minimal inhibitory concentration.

AMB		ITZ		PCZ		VCZ		AND	
Range tested	MIC	Range tested	MIC	Range tested	MIC	Range tested	MIC	Range tested	MIC
0.03-16 μg/mL	1 μg/mL	0.03-16 μg/mL	>16 μg/mL	0.03-16 μg/mL	2 μg/mL	0.03-16 μg/mL	>16 μg/mL	0.03-16 μg/mL	≤0.03 μg/mL

## 3 Discussion

In this case report we describe a *R. argillacea* infection in veterinary medicine in a 4-year-old female intact German shepherd dog. This data matches with data from literature about systemic mycotic infections in general and *R. argillacea* infections in particular. A study with 30 dogs with systemic aspergillosis, which is the most common disseminated mycosis, showed that German Shepherds and female dogs were vastly overrepresented (67% and 77% each) and the median age was 4.5 years [19]. In the previous case reports of *R. argillacea* infections the majority of dogs were German shepherds or German shepherd crosses (10/15) and most of the patients were young adult (not all age data was available). In these cases, there was no evidence of a predisposed sex (8 female vs. 7 male) [[6], [7], [8], [9], [10], [11], [12], [13]]. The reason why German shepherd dogs are prone to fungal infections has not yet been clarified. It has been proposed that the breed has an increased risk of IgA deficiency [15]. In the veterinary *R. argillacea* cases, only one dog was tested for IgA levels and this was in reference range [8].

The infection in this dog was disseminated and could be detected in abdominal and thoracic lymph nodes, spleen, liver, kidneys, pancreas and one eye. All of the previous described veterinary cases were disseminated infections [[6], [7], [8], [9], [10], [11], [12], [13]], which differs from human patients, which mostly show pulmonary disease and less frequently dissemination [1]. Chronic colonization of the airways is also described in cystic fibrosis patients [[2], [3], [4],^14^]. The dissemination to lymph nodes, kidneys, spleen and liver is commonly found in the veterinary cases [6,7,[9], [10], [11], [12], [13]]. Dissemination to the eyes has only been reported in two previous cases so far. In these cases, dissemination to the brain could also be proven, which may indicate neurogenic spreading from the brain to the eye or vice versa [6,10]. In our case, histopathology revealed endophthalmitis of one eye, but no involvement of the brain. Since no adjacent organs were affected, a hematogenous spread is to be assumed. Discospondylitis has been described in 2/3 of cases [[6], [7], [8], [9], [10], [11], [12], [13]], making it one the most common manifestation, but could not be found in this case. Shedding of fungal hyphae via urine was described in about half of the reported cases [6,8,10], but could not be proven in our case due to a negative mycological culture. The largest case series of *R. argillacea* spp. infections in dogs showed fungal growth from the urine in 5/7 cases [10].

Systemic aspergillosis was assumed due to the fungal hyphae in the lymph node and the breed predisposition. Although histopathology demonstrated narrow hyphae in line with aspergillosis, the pleomorphic fungal elements, including forms not typically seen in aspergillosis, argue for molecular identification of causative agents from tissue samples in invasive fungal infections to guide therapy. In the previous mentioned case series of systemic aspergillosis in dogs, itraconazole was the most common used drug [19]. Because of this, itraconazole was chosen in this case too. After two weeks of therapy the abdominal lymph nodes were still severely enlarged and there was new involvement of one eye and the gallbladder, which suggests a poor response. Because there was no growth of fungal cultures from the urine and lymph node FNA samples, antifungal susceptibility testing could not be performed. As the diagnosis of *R. argillacea* infection was made post mortem, the antifungal susceptibility profile of this pathogen was not taken into account in the selection of antifungal agents. The previous veterinary cases showed good susceptibility to itraconazole in most cases [6,8,10,11]. In one case the fungus was minimally resistant to itraconazole [7]. The data in human medicine is also limited. One study showed that *R. argillacea* species had variable susceptibility profiles to itraconazole [18]. The global guidelines for the diagnosis and management of rare mould infections suggest a first-line therapy with caspofungin or micafungin or a combination of one of these with liposomal amphoterecin B (L-AmB) or posaconazole. Azole monotherapy should be avoided [20]. It is not known whether these guidelines can also be applied to dogs. In this case the owner declined intravenous antifungal therapy, so that echinocandins and amphotericin B were no option. Changing treatment to another azole may have prolonged survival due to the described variable susceptibility profiles in human research [18], but the owner selected euthanasia because of ongoing clinical deterioration. Post mortem antifungal susceptibility testing revealed that the pathogen was resistant to itraconazole.

Most of the clinical samples of *R. argillacea* complex in human medicine were obtained through sputum, bronchial aspirates or lung biopsies, which suggests that uptake of the fungus happens through inhalation [2]. This is supported by the facts that about 90% of invasive infections are sited in the lungs of human patients [1] and that cystic fibrosis patients are prone to colonization and infection by *R. argillacea* species [[2], [3], [4],^14^]. It is described that *R. argillacea* spp. infections can spread from the lung to adjacent organs and bones, such as the ribs [1,2,5]. In veterinary cases, it is notable that infections of the thoracic and cranial lumbar vertebral bodies, as well as the sternebrae, were frequently observed [[6], [7], [8],^10^,^11^]. This could suggest that pathogen uptake also happens through inhalation and subsequently spreading towards adjacent bones follows. However, lung involvement is only described in about half of the cases [6,7,9,10,13], so that it remains mainly a speculation from human medicine knowledge that *R. argillacea* spp. is an airborne pathogen. In the veterinary cases of systemic aspergillosis, the site of entry is also unknown, but is suspected to occur through respiratory or gastrointestinal routes. However, only a third of these patients actually show diseases of the associated organ systems, which is comparable to the *R. argillacea* spp. cases [19]. In our case the thoracal lymph nodes showed invasion with fungal hyphae, but this also applied to the abdominal lymph nodes. However, no lesions could be detected in the lungs, so that the entry point of the pathogen remains unclear. *R. argillacea* spp. show a worldwide distribution [18]. It is not yet known where patients take up the pathogen. Other types of fungi that cause infections are found, for example, in houseplant soil [2].

Definitive diagnosis and differentiation of the fungus is usually only possible by DNA sequencing, as *R. argillacea* spp. show morphological similarities to *Paecilomyces* and *Penicillium* spp. [1,2,18]. In the largest case study in human medicine, the misdiagnosis rate before sequencing was about 50%. In most of the veterinary case reports, macroscopic and microscopic examination also led to the suspicion of *Paecilomyces* or *Penicillium* spp [[6], [7], [8], [9], [10],^12^,^13^].

9 of the previous 15 cases belong to *R. piperina* infections [6,9,10,13,18] and the remaining 6 cases are described as *R. argillacea* [7,8,[10], [11], [12]]. In the latter cases, however, it is not always clear whether the classification was made down to the species level or whether only the *R. argillacea* spp. complex was meant. In the field of human medicine, *R. argillacea* is the most common pathogen responsible for invasive infections [1]. Moreover, it is frequently identified as a colonizer in the airways of cystic fibrosis patients, though it is less prevalent than *R. aegroticola* [4,14,18]. The owner of the dog in this case is not aware of anyone in the surrounding who could have been a possible carrier, so the source of infection remains unclear.

The overall prognosis for *R. argillacea* infections in dogs is poor. A complete cure could not be achieved in any case [[6], [7], [8], [9], [10], [11], [12], [13]]. The survival times vary widely from 0 to 24 Months (676 days). About half of the dogs (7/15) died or were euthanised within their first hospitalisation [9,10,12,13]. Four of these dogs died or were euthanised even though they received antifungal therapy. In the biggest case series median survival times were noteable higher in dogs surviving to discharge (6 days vs. 317 days) [10]. Survival times for systemic aspergillosis in dogs are comparable (0-25 months). It was found that dogs displaying less severe symptoms had longer survival times following antifungal therapy [19]. This seems to be comparable to the cases of *R. argillacea* spp. infections. The mortality rate in human medicine is significantly lower (around 40%), possibly because disseminated infections occur much less frequently (30%) [1] in relation to veterinary cases (100%) [[6], [7], [8], [9], [10], [11], [12], [13]]. Our patient was euthanised 29 days after initial presentation.

This is the second case report of a *R. argillacea* spp. infection in a dog in Germany. Overall systemic mycoses are a rare disease in veterinary medicine and *R. argillacea* spp. is an extremely rare pathogen. Due to its rarity, it is unrealistic to expect all practitioners to be aware of this pathogen. It is therefore even more important to increase pathogen differentiation by DNA sequencing in mycology to enable accurate diagnosis and appropriate antifungal therapy. Sequencing should at least be performed if the type of fungus cannot be clearly determined by phenotype and microscopy. Especially in German Shepherds and German Shepherd mixes, fungal infections should be considered as a cause of unclear clinical symptoms and pyogranulomatous inflammation. As *R. argillacea* has already been described as an emerging pathogen in the last decade, it remains to be seen whether this thermophilic pathogen will occur more frequently due to climate change in the next years.

## CRediT authorship contribution statement

**Louisa Schmidt:** Conceptualization, Data curation, Formal analysis, Visualization, Writing – original draft. **Sebastian Reusch:** Funding acquisition, Investigation, Visualization, Writing – original draft, Writing – review & editing. **Ilka McCormick-Smith:** Formal analysis, Investigation, Resources. **Jasmin Gerkrath:** Investigation, Resources. **Martin Peters:** Investigation, Project administration, Visualization, Writing – original draft, Writing – review & editing. **Volker Rickerts:** Investigation, Writing – review & editing.

## Ethical form

Please note that this journal requires full disclosure of all sources of funding and potential conflicts of interest. The journal also requires a declaration that the author(s) have obtained written and signed consent to publish the case report/case series from the patient(s) or legal guardian(s).

The statements on funding, conflict of interest and consent need to be submitted via our Ethical Form that can be downloaded from the submission site www.ees.elsevier.com/mmcr. **Please note that your manuscript will not be considered for publication until the signed Ethical Form has been received.**

## Declaration of generative AI and AI-assisted technologies in the writing process

During the preparation of this work the authors used deepl.com in order to improve readability and language. After using this tool/service, the authors reviewed and edited the content as needed and take full responsibility for the content of the publication.

## Funding

The publication fee was covered by 10.13039/501100023448Robert Koch-Institute, Berlin, Germany. No other funding has been received for this research.

## Conflict of interest

The authors have no conflict of interest to disclose.
